# Evaluation of a polarization-enhanced laparoscopy prototype for improved intra-operative visualization of peritoneal metastases

**DOI:** 10.1038/s41598-023-41361-5

**Published:** 2023-09-09

**Authors:** Thomas Schnelldorfer, Einstein Gnanatheepam, Robert Trout, Ahmed Gado, Joyce-Ellen Pelletier, Long T. Dinh, Martin Hunter, Irene Georgakoudi

**Affiliations:** 1 Division of Surgical Oncology, Tufts Medical Center, 800 Washington St, Boston, MA 02111 USA; 2 Department of Translational Research, Lahey Hospital and Medical Center, 31 Mall Road, Burlington, MA 01805 USA; 3 Department of Biomedical Engineering, Tufts University, 200 College Avenue, Medford, MA 02155 USA; 4 Department of Biomedical Engineering, Duke University, 101 Science Drive, Durham, NC 27708 USA; 5 Google LLC, San Francisco, CA 94105-1673 USA; 6 Department of Biomedical Engineering, S684 LSL, University of Massachusetts at Amherst, 240 Thatcher Road, Amherst, MA 01003 USA; 7 Genetics, Molecular and Cellular Biology Program, Graduate School of Biomedical Sciences, Tufts University, Boston, MA 02111 USA

**Keywords:** Medical research, Oncology, Engineering

## Abstract

Despite careful staging, the accuracy for preoperative detection of small distant metastases remains poor, creating a clinical need for enhanced operative staging to detect occult peritoneal metastases. This study evaluates a polarization-enhanced laparoscopy (PEL) prototype and assesses its potential for label-free contrast enhancement of peritoneal metastases. This is a first-in-human feasibility study, including 10 adult patients who underwent standard staging laparoscopy (SSL) for gastrointestinal malignancy along with PEL. Image frames of all detectable peritoneal lesions underwent analysis. Using Monte Carlo simulations, contrast enhancement based on the color dependence of PEL (mPEL) was assessed. The prototype performed safely, yet with limitations in illumination, fogging of the distal window, and image co-registration. Sixty-five lesions (56 presumed benign and 9 presumed malignant) from 3 patients represented the study sample. While most lesions were visible under human examination of both SSL and PEL videos, more lesions were apparent using SSL. However, this was likely due to reduced illumination under PEL. When controlling for such effects through direct comparisons of integrated (WLL) vs differential (PEL) polarization laparoscopy images, we found that PEL imaging yielded an over twofold Weber contrast enhancement over WLL. Further, enhancements in the discrimination between malignant and benign lesions were achieved by exploiting the PEL color contrast to enhance sensitivity to tissue scattering, influenced primarily by collagen. In conclusion, PEL appears safe and easy to integrate into the operating room. When controlling for the degree of illumination, image analysis suggested a potential for mPEL to provide improved visualization of metastases.

## Introduction

Successful treatment of cancers requires optimal selection of treatment modalities. Surgical resection is a common treatment modality for various cancers and is clinically utilized in almost half of all cancer patients^1^. The appropriateness of surgical resection is typically determined by the extent of the cancer (i.e. staging). Despite careful preoperative radiographic and intra-operative evaluation, the accuracy of staging for distant metastases remains poor, as demonstrated by a significant rate of early cancer recurrence in patients who underwent a “complete” operative resection for many types of cancers^2^. It is therefore desirable to develop an imaging system that will enhance the visibility of these occult and presumed small distant metastases, i.e. increase their optical contrast. For abdominal cancers, this would particularly involve the peritoneal cavity. A label-free optical imaging approach would be well suited for this purpose as it would be easy to integrate with white light laparoscopy, which is the current standard of care. In addition, it would obviate the need for an exogenous contrast agent that would require FDA approval and possibly additional time and/or procedures for contrast delivery and optimal uptake^3–5^. However, in principle the contrast of label-free images tends to be weak and requires more careful processing to extract signals that provide high levels of diagnostic accuracy^6,7^.

We recently reported that differences in the rate of depolarization of linearly polarized incident illumination as detected by a polarization-enhanced laparoscope (PEL) could provide enhanced contrast for changes in collagen fiber organization and cross-section relative to white light laparoscopy (WLL)^8^. This is relevant for this task, since remodeling of the collagen stroma and especially enhanced levels of alignment in malignant versus healthy tissues have been reported consistently for ovarian, pancreatic, and colorectal cancers, which often metastasize to the peritoneum^9–19^. The very limited studies that have assessed the collagen organization of metastases indicate that they exhibit similarities in terms of collagen content and alignment with the primary lesions^20,21^. While such organizational changes may not serve as sources of contrast when using standard laparoscopic imaging, they could be exploited by polarization sensitive imaging approaches. In fact, prior polarimetric imaging studies of colon, cervix, oral, breast, and skin tissues indicated that cancer lesions were less depolarizing than healthy^8,22–30^.

The PEL system used in this study relied on tissue illumination with linearly polarized white light and detection of light that is polarized along parallel (co-polarized) and perpendicular (cross-polarized) orientations relative to the incident illumination. The difference of these two linearly polarized images was considered a PEL image, while their sum was considered the corresponding WLL image, which should in principle be very similar to the images acquired during standard staging laparoscopy (SSL). As indicated above, under PEL imaging the tissue surface is illuminated with linearly polarized white light. Light captured by the camera after being scattered only within the superficial tissue scatters relatively few times and thereby maintains to a large extent its incident polarization providing primarily co-polarized signals. Conversely, light scattered within the deeper tissue layers becomes depolarized, as a result of a multitude of scattering interactions during its longer optical path through tissue before being captured by the camera. This type of scattered light, therefore, contains approximately equal level of co- and cross polarized light relative to the incident illumination. These expected differences in the polarization character of light scattered within the superficial and deeper tissues can be exploited to enhance visualization of the surface signal only. This is achieved via the computation of the difference between the co-polarized (“all” of superficial and “half” of deep) and cross-polarized (“half” of deep) signals:

1 $${I}_{\Delta }{= I}_{\parallel }-{I}_{\perp }$$

2 $${I}_{\Delta }{= (I}_{surface}+{\frac{1}{2}I}_{deep})-\left({\frac{1}{2}I}_{deep}\right)$$

3 $${{I}_{\Delta }= I}_{surface}$$

This type of polarization-gated imaging has been shown to capture a light signal with a very short penetration depth of three optical depths in scattering media (typically on the order of 100–300 μm^26^. Since the penetration depth of white light exceeds the thickness of the peritoneum, this could be advantageous for highlighting changes in scattering properties resulting from the presence of peritoneal metastases, which are primarily present on the peritoneal surface^21^. In addition, the PEL signal increases as linear depolarization (change in the direction of the incident polarization that results from scattering) and retardance (differences in the propagation speed of the light within birefringent media dependent on its polarization) decreases^32^. This results in highlighting of targets, like malignancies, that contain collagen fibers with lower scattering cross-section and higher alignment than the surrounding healthy tissue^33^. Finally, PEL imaging with standard RGB cameras can exploit color sensitivity to enhance visualization of features with absorbance contrast such as vasculature^34^, while enabling the acquisition of regular color unpolarized images (provided by the sum of the two orthogonal polarization images).

We therefore hypothesized that PEL may improve identification of metastases compared to standard laparoscopy during routine abdominal cancer operations based on polarization signatures of peritoneal lesions. After initial studies were performed using in-vitro phantoms and ex-vivo tissues, this study was conducted to evaluate the use of PEL as a novel adjunct to SSL as the first-in-human feasibility study to assess its potential for label-free contrast enhancement of peritoneal metastases. The aim of this early feasibility clinical device study was to evaluate the device design with respect to initial clinical safety, device functionality, and integration into the operating room.

## Results

### Study demographic

Ten patients who underwent staging laparoscopy for gastrointestinal malignancies were included in this study. Of these patients, four were subsequently excluded due to imaging failures [research device not providing adequate imaging due to condensation of the front-end optical window during the operation (n = 3), research device failed pre-procedure tests (n = 1)]. Therefore six patients were included in the analysis. The study group consisted of three patients with pancreatic ductal adenocarcinoma, two patients with gastric adenocarcinoma, and one patient with jejunal adenocarcinoma. This included three men and three women, representing five white and one black individual. The median age was 77 years (range 64 to 96 years). Each patient underwent between one and three biopsies of clinically suspicious appearing peritoneal lesions. One of the six patients had biopsies confirming the presence of peritoneal metastases. The remaining five patients did not show detectable evidence of metastatic disease at the time of operation, nor any radiographic evidence of metastases within 6 months of clinical follow-up.

### Device performance and image quality selection

While PEL images were as expected darker than SSL images, the subjective determination of the effectiveness of illumination of the abdominal cavity and visualization of intra-peritoneal organs demonstrated good visualization in two patients [median image pixel intensity 23.3 (range 7.5 to 42.9), including the patient with peritoneal metastases], moderate visualization in one patient [median image pixel intensity 6.1 (range 0.0 to 7.8)], and poor visualization in three patients [median image pixel intensity 0.0 (range 0.0 to 1.8)]. Data from the latter patients were excluded. Besides limited illumination using a standard light source, fogging of the window on the distal cap was at times an issue, but was well controlled in the 6 study patients. Issues with co-registration of the co- and cross-polarized images, which were a result of the temporal difference in capturing the two images, resulted at times in mild flickering of the PEL video and false borders around the lesion. This was particularly present if the laparoscope moved fast. The safety of the device was good without any research associated complications. It was very well integrated into the operating room environment.

Sixty-five lesions from three patients represented the study sample (9 presumed malignant lesions, 56 presumed benign lesions). Among them, five lesions were biopsied and three of the biopsied lesions were confirmed as malignant (all 3 from one patient) and two of them as benign (from two patients).

### Clinical lesion detection by the surgeon

**Figure 1:**
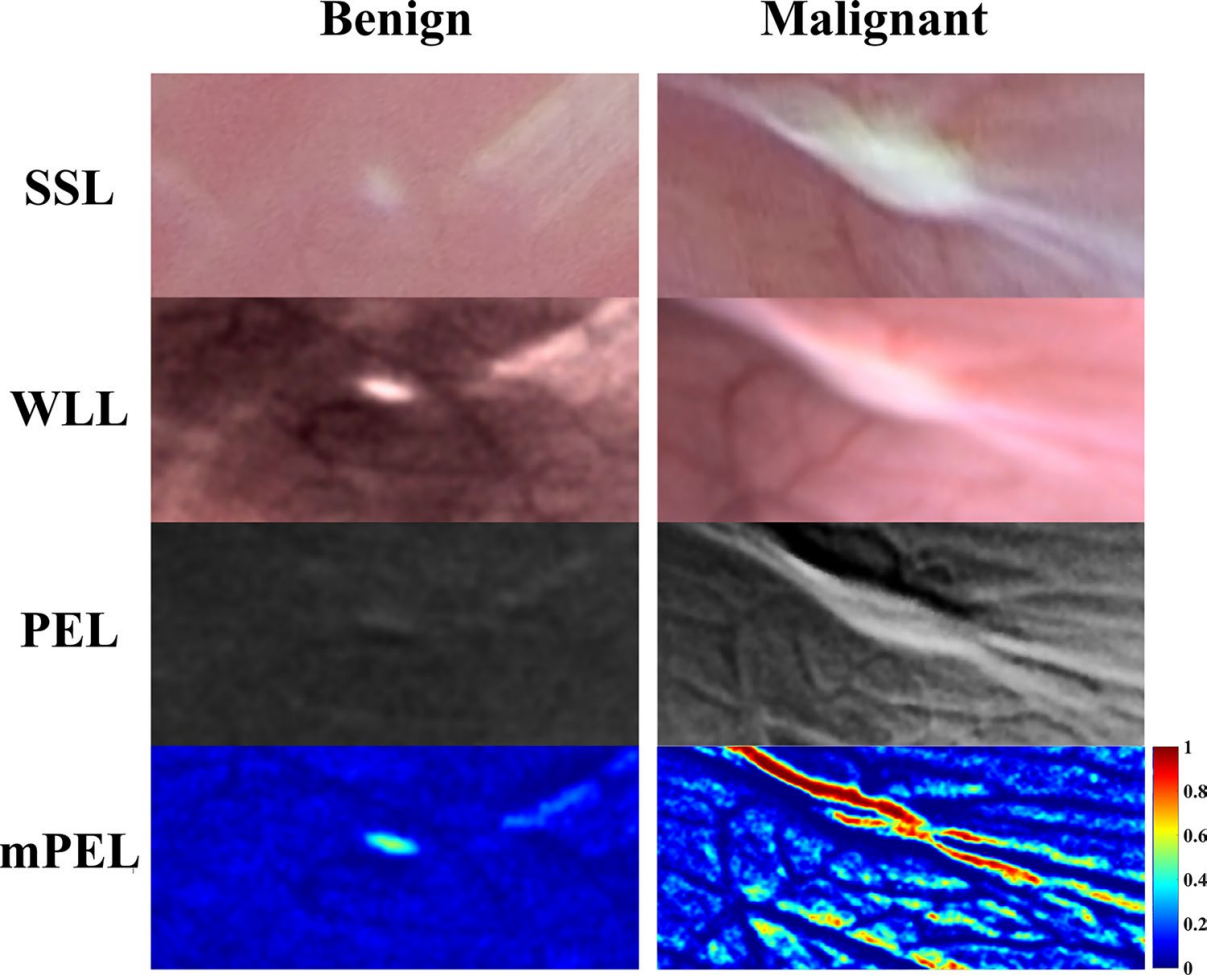
Representative SSL, WLL, PEL, and mPEL images of biopsy confirmed benign lesions and parietal peritoneal metastasis. SSL, WLL, and PEL images are shown as RGB images, while the mPEL images are displayed in pseudocolor (jet map).

Of the 65 study lesions identified post-hoc: (a) 18 (28%) were better seen by the surgeon on SSL [lesion was noted on PEL only after location was identified on SSL (n = 7) or was not seen on PEL at all (n = 11)], (b) 35 (54%) were equally visible with both modalities, and c) 12 (18%) lesions were better seen on PEL [lesion was noted on SSL only after location was identified on PEL (n = 10) or was not seen on SSL at all (n = 2)] (Table 1). Thus, based on visual inspection lesions were visualized with greater sensitivity using SSL than PEL. Examples of the optical appearance of these images are shown in Fig. 1.

**Table 1:** Number of lesions identified during clinical lesion detection (n = 65).

	SSL	PEL	p-value*
Seen in 1st round	53 (82%)	47 (72%)	0.298
Seen in 2nd round (in retrospect)	10 (15%)	7 (11%)	0.604
Not seen	2 (3%)	11 (17%)	0.016

*p-value comparing both methods, Fisher’s exact test.

### Computation of mPEL

To take advantage of color dependent contrast that may enhance sensitivity to collagen structural changes under PEL^36^, Monte Carlo simulations led to the following relationship to optimize the correlation between the potential range of PEL signals detected in each of the camera’s RGB channels and the tissue scattering power (defined as the exponent describing the wavelength dependence of the reduced scattering coefficient, μ_s'_(λ)$\sim {\lambda }^{-b}$):

4 $$mPEL= 0.4705 - 0.0073 \left({R}_{\parallel }-{R}_{\perp }\right)-0.0039 \left({G}_{\parallel }- {G}_{\perp }\right)+0.1104 \left({B}_{\parallel }- {B}_{\perp }\right)$$

**Figure 2:**
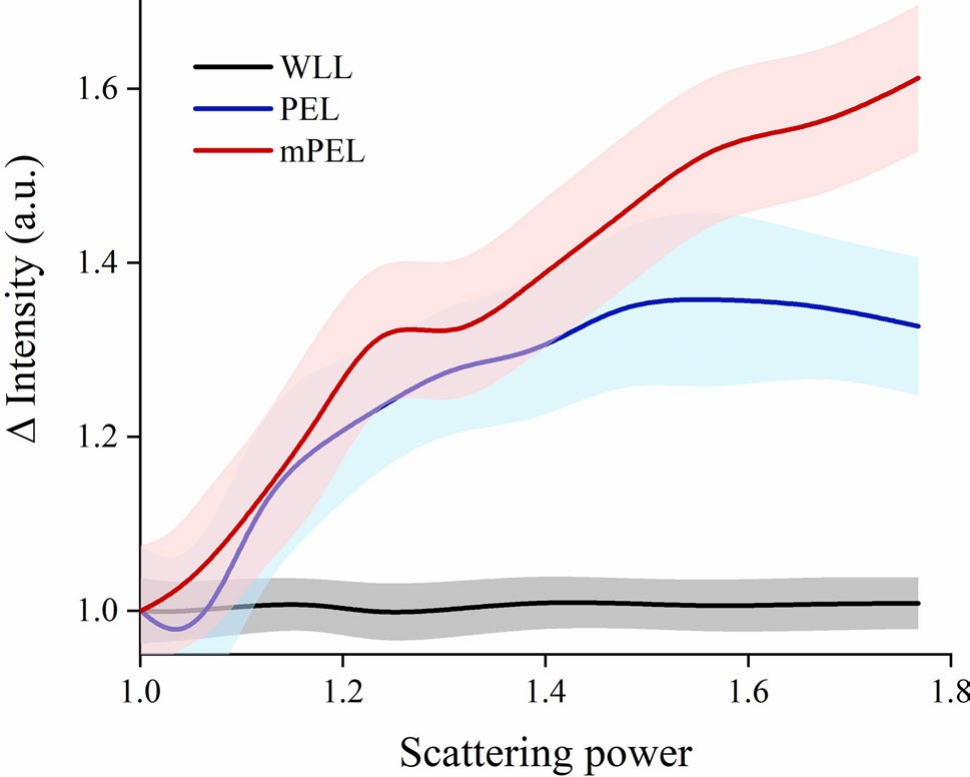
Monte Carlo simulations of WLL, PEL, and mPEL for a range of scattering powers encountered in peritoneal tissue (shaded region represents range of values for different scatterer and hemoglobin concentrations; note that scattering power is a unitless parameter).

This equation was used to create images that represented a modification from the original PEL images (i.e. mPEL). Figure 2 shows Monte Carlo simulations of the relative change in the reflected light intensity as a function of scattering power under WLL, PEL, and mPEL imaging. It is observed that while WLL is independent of scattering power, PEL shows a strong dependence on scattering power, while mPEL exhibits an even stronger dependence than PEL.

Changes in collagen architecture in malignant tissue affect both the reduced scattering coefficient and the scattering power. Scattering power is a reliable parameter for clinical imaging for two reasons. First, scattering power is independent of the illumination intensity and the collection efficiency of the imaging system^48^. This is because the scattering power relates to the slope of the wavelength-dependent reduced scattering coefficient. In addition, the scattering power is strongly correlated with the effective scattering cross section of tissue^49–51^. Since collagen is a major contributor to the effective scattering cross-section, modalities such as mPEL that are sensitive to scattering power changes can potentially probe the structural and organizational changes in collagen that are often present in malignant tissues.

### Digital lesion detection

**Figure 3:**
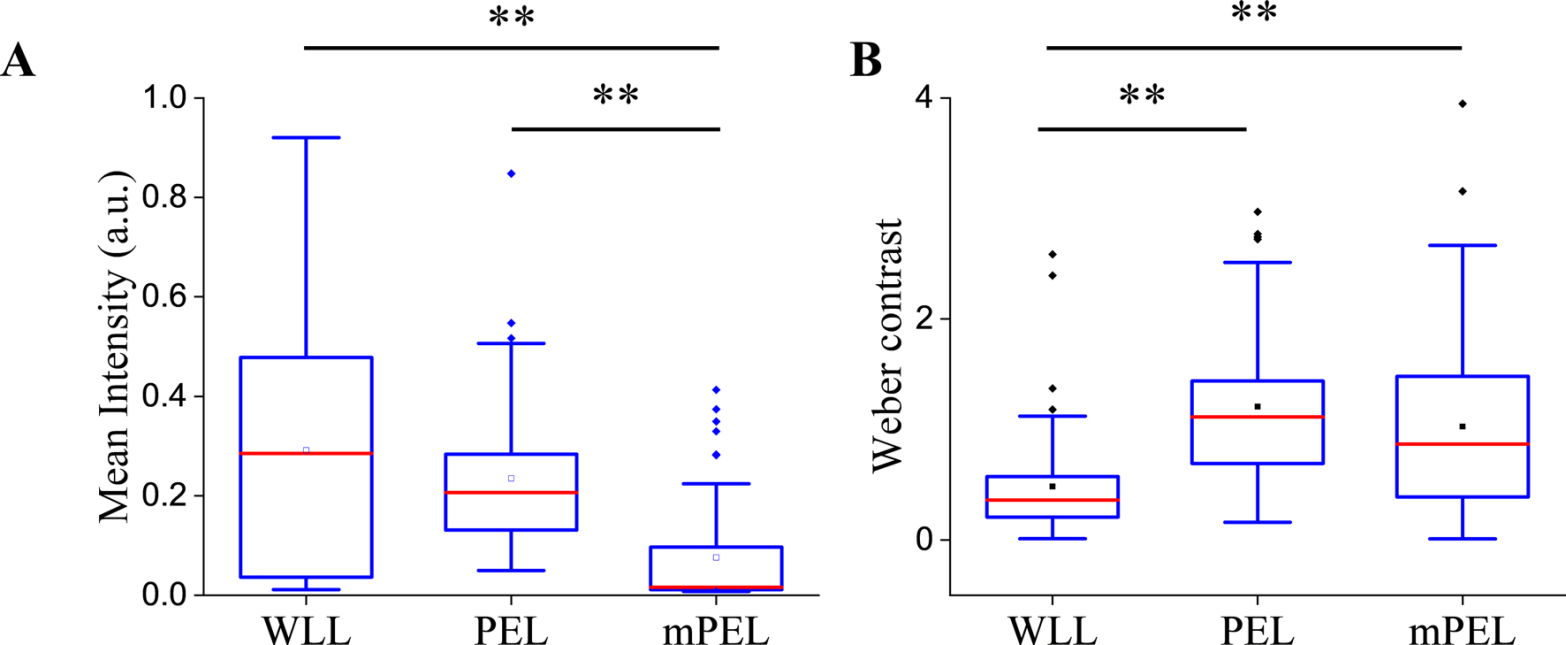
Mean ROI intensity (**A**) and Weber contrast (**B**) for all 65 study lesions in gray channel under each imaging modality. Statistical significance was determined by one-way ANOVA with Tukey's HSD post-hoc tests. ** denotes p-value < 0.001.

Assessing all 65 study lesions, the mean ROI total signal intensity (sum of red, blue, and green channels) under PEL and mPEL was 1.3-fold and 3.8-fold lower compared to WLL, respectively (Fig. 3A). The Tukey’s post hoc test confirmed that there was significant difference between the mean intensity of WLL and mPEL images (p < 0.001), but the difference between WLL and PEL was insignificant (p = 0.186). Yet, due to the overproportionate darker background on PEL, the corresponding mean Weber contrast under PEL was 2.5-fold greater compared to WLL, suggesting the potential for improved lesion detection using PEL when controlled for the degree of illumination (p < 0.001). Upon exploiting the color-dependence of PEL, mPEL provided 2.1-fold greater Weber contrast compared to WLL (p < 0.001, Fig. 3B).

### Digital lesion classification

**Figure 4:**
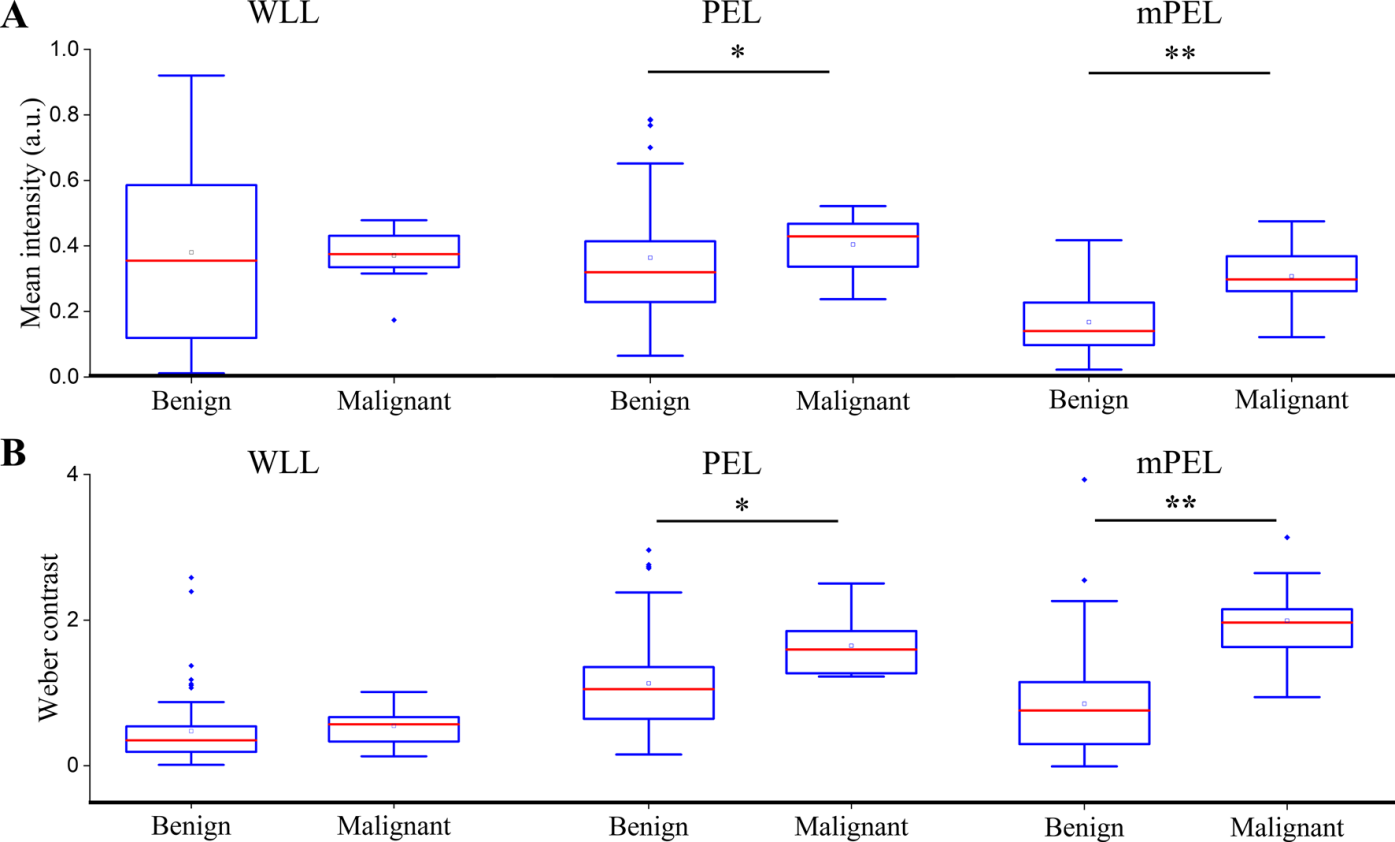
Box plots of (**A**) mean intensities and (**B**) Weber contrast of presumed-benign (n = 56) compared to presumed-malignant (n = 9) lesions in different imaging modalities. * denotes p-value < 0.05. ** denotes p-value < 0.001.

There was no measurable difference in ROI intensity when comparing presumed benign lesions (n = 56) to presumed malignant lesions (n = 9) observed under WLL (p = 0.910). However, PEL and mPEL were able to provide a 1.1-fold and 1.8-fold greater ROI intensity in presumed malignant lesions compared to presumed benign lesions, respectively (PEL p = 0.037, mPEL p < 0.001, Fig. 4A). The findings indicate that malignant lesions are characterized by a higher scattering power than benign lesions (mean malignant mPEL intensity = 0.30 ± 0.10; mean benign mPEL intensity = 0.16 ± 0.09).

It is theoretically possible that these findings might be due to differences in illumination, since all presumed malignant lesions came from a single patient. Nevertheless, Weber contrast confirmed the same findings making illumination differences less likely. Specifically, the Weber contrast of PEL and mPEL images was 1.4-fold and 2.3-fold greater than WLL images for malignant compared to benign lesions, respectively (PEL p = 0.031, mPEL p < 0.001, Fig. 4B).

### Correlation of mPEL to collagen structure

**Figure 5:**
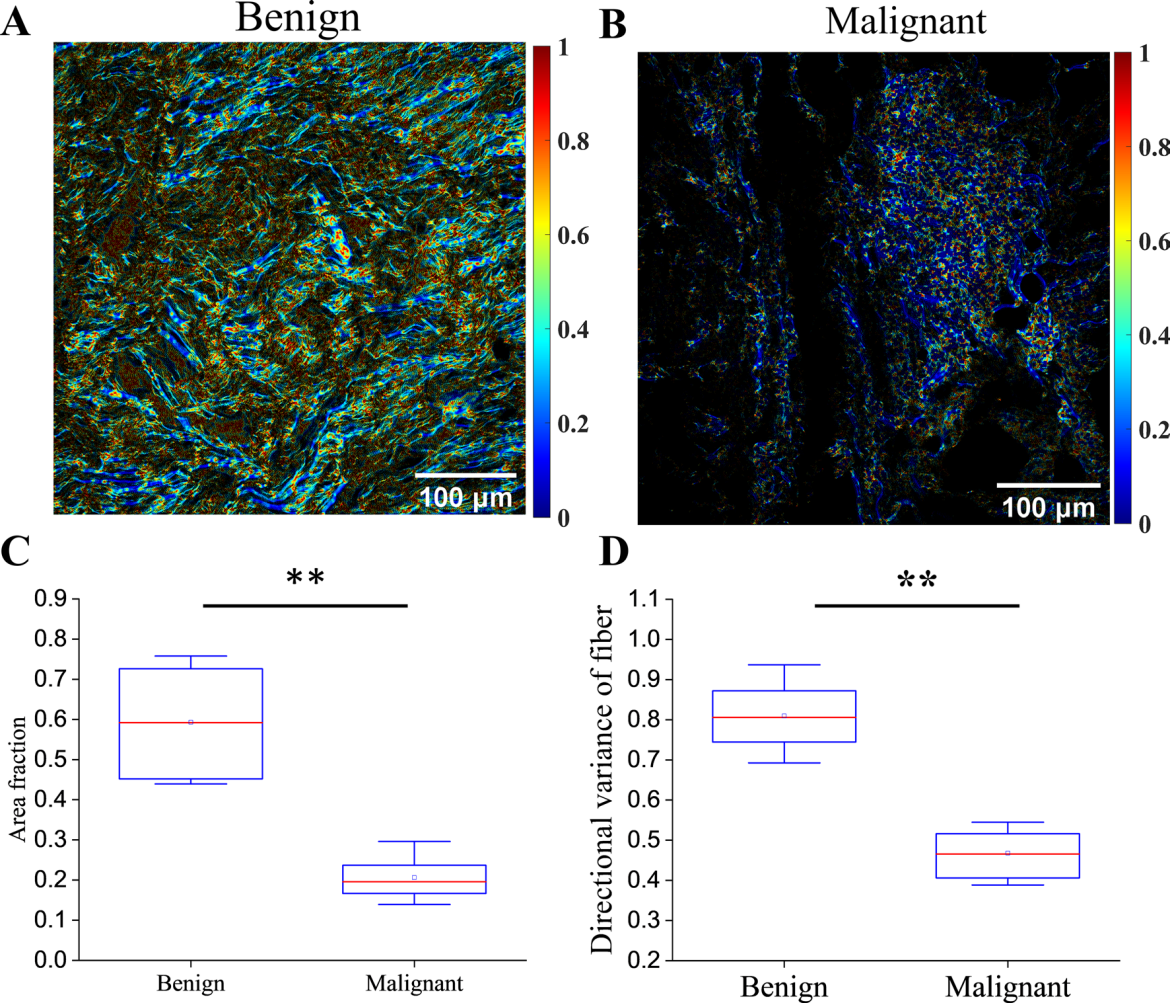
2D variance of collagen fibers of (**A**) malignant and (**B**) benign lesions. Box plot of volume fraction (**C**) and mean directional variance of fibers (**D**) for malignant and benign lesions. ** denotes p-value < 0.001.

To better understand the etiology of the augmented signal between presumed malignant lesions to presumed benign lesions provided by mPEL, microarchitectural changes in the collagen organization of benign and malignant lesions were assessed in all available biopsy samples from this study. Specifically, SHG images were acquired from two benign and three malignant lesions (Fig. 5A, B). Three ROIs from each benign and malignant lesion were imaged. The analysis results showed that there is lower collagen volume fraction in malignant lesions compared to the benign ones (Fig. 5C). Further, the directional variance of the collagen fibers within the malignant lesions was found to be lower than that of the benign lesions (Fig. 5D).

## Discussion

This early feasibility study demonstrated that laparoscopy using the designed PEL prototype can be performed safely in patients undergoing staging laparoscopy. We identified areas that can benefit from future improvement of the device, including increasing the intensity of illumination, avoiding front-end optical window condensation, and modifying the image analysis software to address issues with co-registration and to take advantage of wavelength-dependent differential polarization backscattering (mPEL). Reasonable measurements on the visualization of peritoneal metastases under PEL and mPEL in comparison to WLL/SSL were achieved (Fig. 1).

Subjective detection of peritoneal lesions by a human examiner was seemingly overall worse with PEL compared to SSL. These differences were likely due to significantly darker illumination under PEL compared to SSL, supported by the findings of the digital analysis demonstrating lower lesion intensity values under PEL. However, using Weber contrast as a means to digitally control for the degree of illumination, improved lesion detection was noted with a 2.5-fold increase in contrast and therefore potential for better visualization of any lesion using PEL compared to WLL. This enhancement occurs because under PEL imaging the background is suppressed at higher levels than the signal emanating from the superficial lesion containing regions. The effect was similar when using mPEL providing a 2.1-fold increase. The results suggest that with future improvements in illumination using a light source with a higher power rating, PEL and mPEL should be able to significantly enhance contrast to theoretically allow for a human to better recognize peritoneal lesions. Specifically, the current imaging quality of the prototype likely was the reason for worse detection by a human examiner. But the improvement in contrast, particularly with mPEL, provides promise for future improved detection by automated systems and if the imaging quality is improved even by a human examiner.

The study further assessed the prototype’s performance in classifying peritoneal lesions. For this task, PEL also seemed to perform better than WLL. It provided a threefold greater Weber contrast for malignant lesions compared to benign lesions. mPEL, with its wavelength-dependent measure, seemed to even further augment this difference (3.6-fold increase). If this trend is validated with a larger scale study, it could be used to achieve enhanced sensitivity and specificity for identifying potentially occult malignant lesions, supporting their usefulness for staging laparoscopy.

The enhanced PEL/mPEL contrast for malignant versus benign peritoneal lesions is consistent with the changes in collagen content and organization observed in this study in fixed sections from benign and malignant biopsied tissues. Our prior studies also indicated that malignant tissues, including peritoneal metastases, harbor a lower volume fraction of more highly aligned collagen fibers compared to healthy tissues^21,33^. These changes are expected to lead to a decrease in scattering cross section, and a subsequent reduction in the depolarization of backscattered light and corresponding increase in the PEL signal^33^. Under mPEL, this phenomenon is enhanced further as we aim to achieve an optimized correlation of the mPEL signal to scattering power.

Our findings are consistent with a number of polarimetric imaging studies indicating that a wide range of cancer tissues are less depolarizing than corresponding healthy tissues^22–30^. Mueller-matrix polarimetric imaging systems that characterize fully the polarization-dependent interactions between light and tissue are likely to yield the most sensitive assessments of tissue scattering changes^22,52–55^. However, the instrumentation, image acquisition, and processing time required for the 16 needed polarization-resolved images limit integration into a real-time surgical guidance workflow, even with recent advances that decrease the acquisition frame rate to as low as 1 s^52,55^. Faster implementations of simpler polarized systems exist, including those which are based on differential polarization^56^. For example, linear, cross-polarized imaging has been used to visualize collagen disruption at the margins of skin cancers during resection^41,57^. Circularly polarized light-based systems were also considered to image subsurface tissue layers, since the rate of depolarization of circularly polarized light is slower than that of linearly polarized light^58,59^. However, since peritoneal lesions are typically around the thin (~ 5 μm) peritoneal lining, linear polarization gating, like PEL, is more suitable for enhanced visualization of superficial changes in the collagen structure. While RGB contrast has been exploited in differential polarization systems to enhance contrast for visualizing blood vessels^52^, and in cross-polarized images to enhance contrast from collagen in deeper layers, our study indicates that in the context of laparoscopy, color sensitivity of differentially polarized light enhances contrast to superficial collagen changes.

This study’s main limitation is decreased image quality in some of the images mostly due to poor illumination. Images with very poor illumination were excluded, presumably without introducing large selection bias. Limitations in other forms of image quality as a result of using a prototype were controlled for in the digital analysis by using WLL rather than SSL as a comparison. Patient sample size was limited, but a reasonable number of lesions were imaged and analyzed. This, however, might limit optical heterogeneity of the lesion sample to some degree given the limited number of patients these lesion images originated from. Analyses were done mostly under a presumed pathology status given the limited number of biopsy-proven pathology amongst all lesions. This provides some potential measurement bias for the classification task. Nevertheless, we note that the assigned “presumed” labels correlated with the patients’ clinical outcome (i.e. none of the patients with presumed benign lesions had radiographic cancer progression within 6 months after the operation and the only patient with presumed malignant lesions had three lesions that were biopsy-proven metastases).

PEL and mPEL have potential to improve detection of peritoneal lesions and to help differentiate benign from malignant lesions. This contrast enhancement likely arises from the changes in collagen content and organization that are present in superficial peritoneal metastatic lesions from most solid cancers^28^. Thus, this study supports continued investigation of these techniques to assess their potential to improve the current limitations of operative identification of occult cancer metastases. Improved understanding of the range of collagen organization changes that may be associated with the primary tumor origin and the impact of such variations on PEL/mPEL vs SSL images would be important for optimizing detection. The ease of integration of PEL/mPEL into routine laparoscopic imaging facilitates the pursuit of such studies and their broader adoption if successful. The next generation prototype could include improvements in illumination, image acquisition speed, and sheath material design to avoid fogging and sealing issues that limited the performance of the original PEL system. Such modifications, would enable simultaneous visualization of WLL and PEL or mPEL images for the surgeon, with WLL images of similar quality to SSL ones. Ultimately, real-time diagnostic algorithms could be implemented based on the mPEL features to guide biopsy. Overall, there seems promise that label-free imaging using information from the polarization status of reflected light during staging laparoscopy would be able to improve operative staging to allow for more optimal selection of treatment modalities for patients with various cancers.

## Methods

### Study population

This first-in-human early feasibility study was approved by the Lahey Clinic Institutional Review Board. Ten adult patients (age ≥ 18 years) who were scheduled to undergo SSL for biopsy-proven malignancy of the gastrointestinal tract at Lahey Hospital in Burlington, MA were recruited. Exclusion criteria included pregnancy, BMI > 60 kg/m^2^, severe cardio-pulmonary comorbidities, coagulopathy, emergent operation, and vulnerable populations. Study patients underwent a written informed consent process. Recruitment was conducted between December 2018 and February 2020. All methods were performed in accordance with the relevant guidelines and regulations.

### Study device

**Figure 6:**
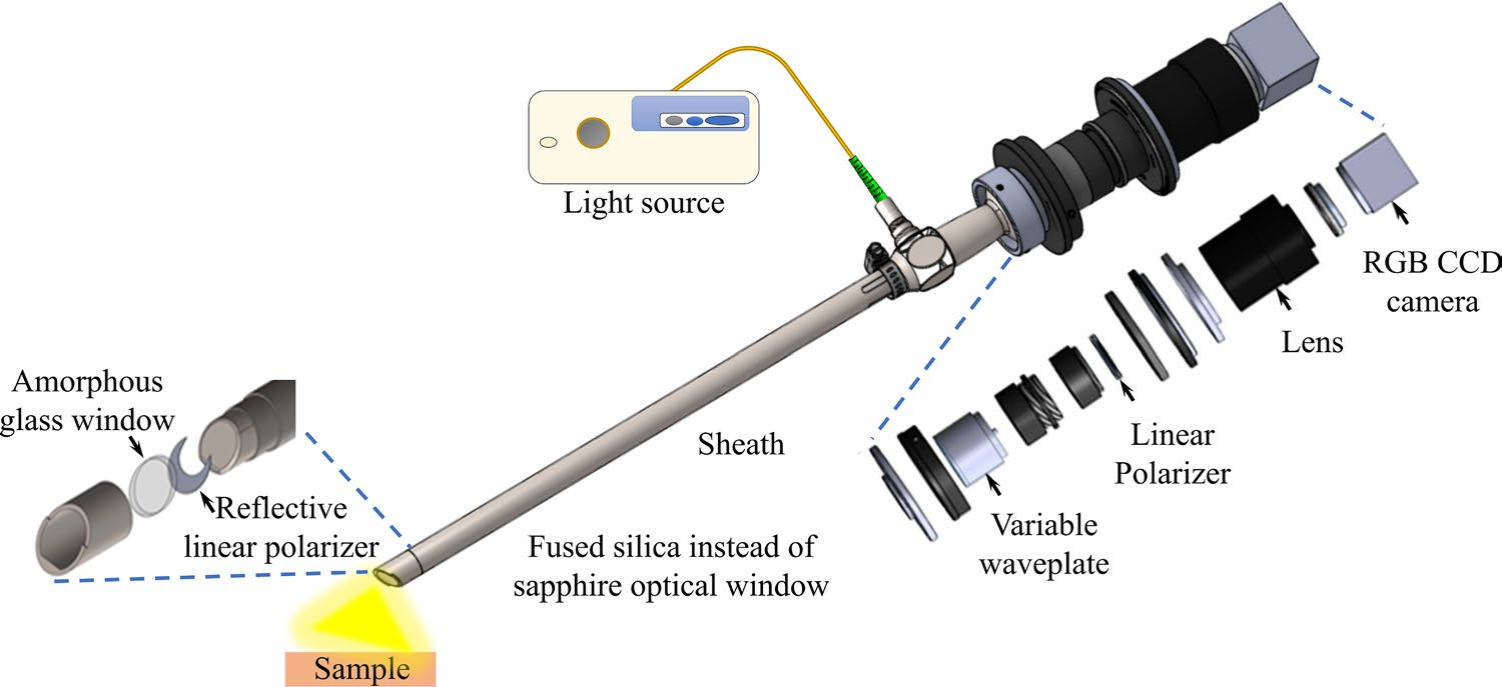
PEL device with proximal optical assembly and distal cap components highlighted in more details.

This study utilized an SSL system and a custom-made PEL prototype. The SSL system consisted of an FDA-approved 10 mm 30-degree rigid laparoscope (Olympus EndoEYE, Center Valley, PA) connected to a standard light source (Olympus Evis Exera II CLV-180) and processor (Olympus Evis Exera II CV-180). The PEL prototype (Fig. 6) consisted of a lens rod (Karl Storz Hopkins II 10 mm 30-degree model 26003BA, Tuttlingen, Germany) that had undergone custom modification to include a fused silica optical window to avoid polarization effects exhibited by the conventional sapphire window^35,36^. The lens rod was connected via a fiber-optical cable to a standard light source (Olympus Evis Exera II CLV-180). Due to the depolarizing effects of the fiber optics, polarization filtering of the illumination had to be conducted at the distal end of the fibers. Therefore, the lens rod was covered by a metal sheath with a cap threaded to the distal end that included a linear polarizing film (Thorlabs LPVISE100-A, Newton, NJ) only covering the illumination aperture but not the camera inlet window (Fig. 6). The setup allowed for linearly polarized illumination.

The resulting reflectance from the abdominal cavity was captured through the aperture of the GRIN lens rod, which was not expected to modify the polarization state of the light. Attached to the proximal end of the lens rod was a mount adapter that connected to an optical assembly to allow for PEL imaging (Fig. 6). Within the assembly, image signals were relayed through an electrically tunable liquid crystal variable wavelength retarder (Thorlabs LCC1111T-A) with the slow axis oriented at 45 degrees. Two operating voltages were switching between zero (1.6 V) and half-wave (25 V) retardance for each frame. In this way, a series of alternating images were created that alternated between an image that maintained the original polarization (zero retardance) and an image that rotated the polarization by 90 degrees (half-wave retardance), respectively. From the retarder, the images traveled through a linear polarizer (Thorlabs LPVISE100-A) that was in line with the illumination polarization to isolate the co- (zero retardance) and cross- (half-wave retardance) polarized components of the collected signal. Subsequently, the images traveled through a varifocal zoom lens (Computar 12–36 mm, Cary, NC) before being captured onto an RGB charge coupled device (CCD) camera with 1920 × 1200 pixel resolution, at a rate of 27 frames per second, and 12-bit pixel depth (FLIR Blackfly 23S2C-CS, Wilsonville, OR). The switching of the retarder was synchronized with the camera's frame collection utilizing a built-in strobe feature. The series of collected images alternating in polarization state were then processed in real-time to generate a new stream of images, with the PEL image computed as the difference of co- and cross- polarized images and the WLL image computed as their sum. Prior to its use in the clinical study, the device underwent laboratory performance evaluations as well as mechanical, electrical, thermal, chemical, and sterilization safety tests.

### Intra-operative imaging

Patients underwent SSL evaluation of the abdominal cavity at the beginning of the operation according to best medical practice immediately followed by PEL. During PEL examination, WLL images ($I={I}_{\parallel }+{I}_{\perp }$) and PEL images (${I}_{\Delta }={I}_{\parallel }-{I}_{\perp }$) were displayed on the operating room monitors. Co- and cross polarized images were separately recorded to allow for post-hoc analyses. Any biopsies of lesions were conducted after completion of PEL under SSL using standard clinical practice. All procedures were performed by a single surgeon (T.S.). After the operation, the recorded videos were analyzed for the effectiveness of illumination of the abdominal cavity and visualization of intra-peritoneal organs along with the clinical safety of the device.

### Image capture and human performance analysis

For quantitative comparison of lesion visibility across each modality, single representative image frames depicting a peritoneal lesion were collected post-hoc from the SSL and the PEL videos by a single surgeon (T.S.). SSL videos were reviewed first. From the videos, any detectable lesion of any of the peritoneal surfaces was captured by a representative still image depicting the lesion. The images were recorded in TIFF format (24-bit RGB, lossless compression). Similarly, PEL videos were separately reviewed and lesion images were captured providing PEL and WLL images. Thereafter, the image collections from SSL and PEL modalities were evaluated to identify any lesions seen in both modalities (i.e. WLL images from the PEL device were compared to SSL images based on surrounding anatomic landmarks).

A second review of each video was performed to determine whether any lesions noted on the opposing modality could in retrospect be detected. Images of lesions identified during the second review were captured. If a corresponding lesion was not identified, the expected region of the lesion was captured using a similar vantage point as the opposing modality. According to the second review, lesions were classified based on whether they were seen equally with both modalities (i.e. both seen during first review), only seen in retrospect once noted with the other modality (i.e. only noted during second review), or not seen with one modality at all. Further, lesions were classified as “presumed benign” if (1) a biopsy of the lesion was performed and deemed benign or (2) if no biopsy of the lesion was performed and based upon clinical judgement the lesion was considered of low probability to be malignant. “Presumed malignant” lesions were determined based on similar concepts.

### Digital image processing analysis

The performance of PEL was subject to issues with image quality, primarily resulting from the reduced illumination power of the prototype, since essentially half of the light was blocked by the illumination linear polarizer. This was expected to bias the comparison analysis of the prototype to the current clinical standard (i.e. SSL from a fully developed system). To mitigate this bias for any digital analyses, we used WLL images defined as the sum of co- and cross-polarized images (i.e. unpolarized light images) obtained from the prototype in lieu of the SSL images. Since WLL captures both surface and subsurface features, WLL images should be in principle very similar to SSL images. WLL and PEL images used in the analysis were computed respectively as the sum and difference of co- and cross-polarized signals:

5 $$WLL={I}_{surface}+{I}_{deep}= \left({R}_{\parallel }+{R}_{\perp }\right)+ \left({G}_{\parallel }+ {G}_{\perp }\right)+\left({B}_{\parallel }+ {B}_{\perp }\right)$$

6 $$PEL={I}_{surface}= \left({R}_{\parallel }-{R}_{\perp }\right)+ \left({G}_{\parallel }- {G}_{\perp }\right)+\left({B}_{\parallel }- {B}_{\perp }\right)$$

where R, G, B refer to red, green, and blue components of white light, respectively, and the ‖, ⊥ subscripts refer to co- and cross-polarized components of light, respectively.

**Figure 7:**
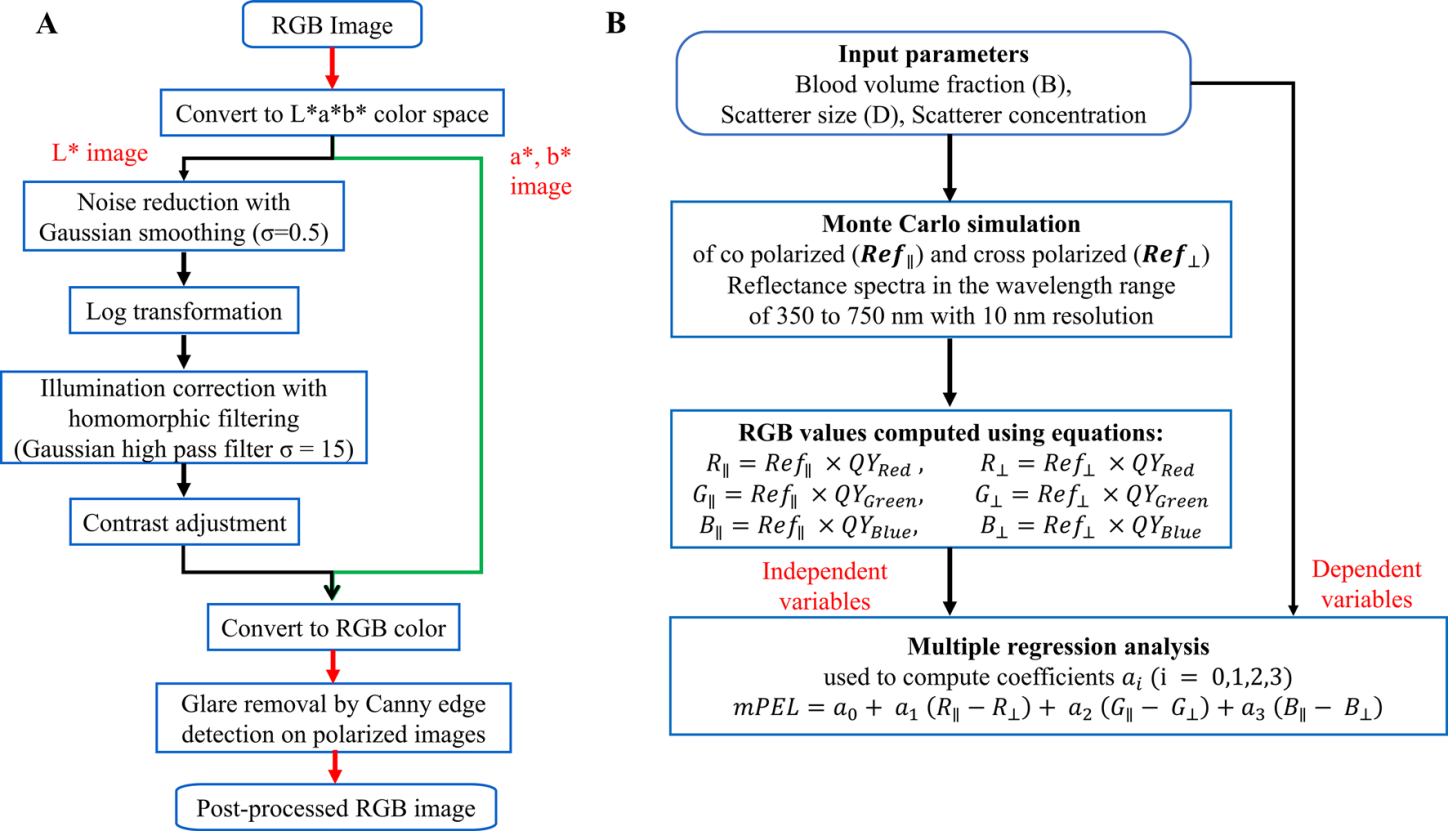
Flow chart of image pre-processing methods (**A**) and computing modified PEL using Monte Carlo-based regression analysis (**B**).

All WLL and PEL study images were pre-processed to reduce detector noise and to enhance contrast using MATLAB software version R2019a (MathWorks, Natick, MA) (Fig. 7A). Original images were converted from RGB to Lab color space where image L contained luminosity and images a and b contained information of green–red and blue-yellow spaces respectively. We applied the following processing protocol on L, while images a and b were unmodified: Gaussian smoothing to reduce detector noise (2-D Gaussian smoothing kernel with standard deviation of 0.5), homomorphic filtering to address non-uniform illumination artefacts (low frequency bands were filtered out from log-transformed images using a Gaussian filter with standard deviation of 15), and contrast adjustment (saturating the bottom 1% and the top 1% of all pixel values)^37^. The resulting Lab color space image was converted back to RGB color space. Further, glare regions were identified based on a Canny edge detection algorithm applied to pixels representing the upper 0.5^th^ intensity percentile of the co-polarized images and the lowest 0.5^th^ intensity percentile of the corresponding cross-polarized images, and removed.

Regions of interest (ROIs) were manually outlined on WLL and PEL images containing the visible borders of the lesions. In case a lesion was only visible in the opposing modality, the expected region of the lesion based on anatomic landmarks was used as ROI. Mean intensity measures were recorded within the ROI. Weber contrast was computed accounting for the entire surrounding image background other than the ROI, where Weber contrast (C_w_) as a function of intensity in the ROI (I_f_) and intensity of the entire background (I_b_) was defined as:

7 $${C}_{W} = \frac{{I}_{f}-{I}_{b}}{{I}_{b}}$$

### Computation of modified PEL through Monte Carlo-based regression analysis

Since the extent of backscattering under PEL was expected to differ at various wavelengths (colors), a model was developed to account for this wavelength dependence to further augment the sensitivity of PEL to scattering changes, and specifically to the scattering power. In principle, the tissue reduced scattering coefficient can be approximated by $\sim {\lambda }^{-b}$, where $\lambda$ is wavelength, and b is defined as the scattering power. For this purpose, a Monte Carlo-based regression analysis was performed to extract the general scattering power dependence on the three RGB color channel intensities of the PEL images, based on a weighted linear equation (Fig. 7B)^38–41^. Since scattering cross section of tumor tissue is generally lower than that of the surrounding tissue, and scattering power is inversely proportional to scattering cross section, lesions were expected to exhibit a greater scattering power than the background tissue resulting in a positive Weber contrast^33,42^.

Briefly, polarization-sensitive Monte Carlo simulations for a single layered tissue model written in C were used to compute polarized light propagation in tissue with similar optical properties to those of peritoneal tissue^38,39^. The code simulated Mie theory scattering for spherical particles and tracked the polarization direction of light using the Stokes vector. The optical depth of the medium was limited to 20, since the WLL signal saturates for larger thicknesses^31^. Here, optical depth was defined as $\tau =\left({\mu }_{a}+{\mu }_{s}\right)D,$ with D representing the geometrical thickness. The Monte Carlo simulation generated a lookup table for reflectance intensities for a range of reduced scattering coefficients (10 cm^−1^ to 35 cm^−1^ with 5 equal steps), scattering cross sections (0.5 to 8 µm^2^ with a step size of 0.5 µm^2^), and blood volume fractions (0.4 to 0.9% with a step size of 0.1%) that are typically encountered in soft tissue under different pathological conditions^42,43^. Absorption coefficients were estimated by assuming a hemoglobin concentration in blood of 15 g/dL^42^. Extinction coefficients of hemoglobin (${\varepsilon }_{Hb}$) were obtained from the public domain^44^. An anisotropy coefficient of 0.9 for the tissue was used in the simulations^42^. A refractive index of 1.35 and 1.42 were fixed for the surrounding tissue and scatterers in the simulations^45,46^. Finally, RGB values were obtained from the reflectance spectra by considering detector efficiency profiles (QY_red_, QY_green_, QY_blue_) of the digital camera in the wavelength range of 350 to 750 nm.

Multiple regression analysis with simulated data sets, established a regression equation for tissue scattering power^40,41^, as

8 $$mPEL={a}_{0}+ {a}_{1} \left({R}_{\parallel }-{R}_{\perp }\right)+ {a}_{2} \left({G}_{\parallel }- {G}_{\perp }\right)+{a}_{3} \left({B}_{\parallel }- {B}_{\perp }\right)$$

The regression coefficients ${a}_{i} (\mathrm{i }=\mathrm{ 0,1},\mathrm{2,3})$ were estimated in MATLAB using the inbuilt function ‘regress’ and they reflected the contributions of RGB values to scattering power. The resulting images were contrast adjusted by saturating the bottom 1% and the top 1% of all pixel values.

### 2D variance of collagen fibers in biopsied lesions

Hematoxylin and eosin-stained histology slides were obtained from all lesions that were biopsied during the operation and that were included in the analysis (3 malignant and 2 benign). The slides underwent imaging using a laser scanning confocal microscope (Leica TCS SP8, Wetzlar, Germany) equipped with Ti:sapphire laser (Spectra Physics, Mountain View, CA). Second harmonic generation (SHG) images were acquired with an excitation wavelength of 860 nm and recorded at 425 ± 25 nm using a non-descanned detector. Laser light was focused on the sample using a water immersion 25× objective (0.95 numerical aperture) to provide a sufficient field of view of 465 × 465 μm^2^. The biopsy samples had 8 to 10 µm thickness and they were imaged along a z stack with a 2 μm step. Three ROIs from each lesion were imaged. The localized 2D directional variance of the collagen from SHG images were computed using a 2D variance algorithm as previously described^47^. The algorithm segmented the fiber-only regions based on Otsu thresholding and the orientation of the fibers were estimated based on a weighted vector sum approach. The distribution in the azimuthal angles was used to estimate the 2D variance of the collagen in the biopsy tissues. The area fraction of the collagen was estimated as the ratio of the pixels within the segmented fiber-only region to the pixels within the entire field.

### Statistical analysis

Descriptive statistics were obtained, and bivariate analyses were used to assess the relationship between lesion classification and the image signal signature. Mean signal intensities and Weber contrast of lesions in the WLL, PEL, and mPEL images were compared using one-way ANOVA with Tukey's HSD post-hoc tests. The difference in mean intensities and Weber contrast of benign and malignant lesions under WLL, PEL, and mPEL were compared using two sample t-tests. Statistical significance was considered for p < 0.05. All statistical analyses were performed using MATLAB.

## Data Availability

The datasets generated and/or analyzed in this study are not publicly available as the Ethical Review Board has not approved the public availability of these data. Correspondence and requests for materials should be addressed to T.S.
